# A case study on the use of Public Narrative as a leadership development approach for Patient Leaders in the English National Health Service

**DOI:** 10.3389/fpubh.2022.926599

**Published:** 2022-09-15

**Authors:** Emilia Aiello, Kathryn Perera, Mo Ade, Teresa Sordé-Martí

**Affiliations:** ^1^Department of Sociology, Autonomous University of Barcelona, Cerdanyola del Vallés, Barcelona, Spain; ^2^National Health Service (NHS) Horizons, London, United Kingdom; ^3^Maternity Voices Partnership (MVP) Chair and Patient Public Voice, National Health Service, Ashford, United Kingdom

**Keywords:** Public Narrative, leadership, Patient Leaders, maternity services, community

## Abstract

**Background:**

In 2016 the National Health Service (NHS) England embraced the commitment to work for maternity services to become safer, more personalized, kinder, professional and more family-friendly. Achieving this involves including a service users' organizations to co-lead and deliver the services. This article explores how Public Narrative, a framework for leadership development used across geographical and cultural settings worldwide, can enhance the confidence, capability and skills of service-user representatives (or Patient Leaders) in the National Health Service (NHS) in England. Specifically, we analyse a pilot initiative conducted with one cohort of Patient Leaders, the Chairs of local Maternity Voices Partnerships (MVPs), and how they have used Public Narrative to enhance their effectiveness in leading transformation in maternity services as part of the NHS Maternity Transformation Programme.

**Methods:**

Qualitative two-phase case study of a pilot training and coaching initiative using Public Narrative with a cohort of MVP Chairs. Phase 1 consisted of a 6-month period, during which the standard framework was adapted in co-design with the MVP Chairs. A core MVP Chair Co-Design Group underwent initial training and follow-up coaching in Public Narrative. Phase 2 consisted of qualitative data collection and data analysis.

**Results:**

The study of this pilot initiative suggests two main ways in which Public Narrative can enhance the effectiveness of Patient Leaders in service improvement in general and maternity services in specific. First, training and coaching in the Public Narrative framework enables Patient Leaders to gain insight into, articulate and then craft their lived experience of healthcare services in a way that connects with and activates the underlying values of others (“shared purpose”), such that those experiences become an emotional resource on which Patient Leaders can draw to influence future service design and decision-making processes. Second, Public Narrative provides a simple and compelling structure through which Patient Leaders can enhance their skills, confidence and capability as “healthcare leaders,” both individually and collectively.

**Conclusions:**

The Public Narrative framework can significantly enhance the confidence, capability and skills of Patient Leaders, both to identify and coalesce around shared purpose and to advance genuine co-production in the design and improvement of healthcare services in general and maternity services in specific.

## Introduction

*It started well with me … healthy first-time mum … low risk pregnancy … experienced professional, confident, comfortable and pretty much together … albeit way more unprepared that I thought. Then my baby forgot to pay attention to his due date memo; ushering in a cascade of interventions like a pack of falling cards and my descent into a deep dark abyss like none I had experienced. From turning down a sweep at my last midwife appointment, no clue what it meant… to being booked for an induction which I didn't understand … to waiting all day to see the obstetrician, who said: “Your chances of a stillbirth increase by 50% if you don't have the induction”, and eventually to a c-section, then breastfeeding problems ….and so on (…) The themes in my story are not new to you: Ignorance & confusion; the use of big data to softly coerce consent, the early feelings of hope and the later sense of loss … the cries of not being listened to and more. We talk of maternity “transformation” whilst sometimes forgetting that every birth transforms the woman, her family and the professionals who help to make it happen. Maternity transforms regardless, for good or bad. Today, we must work to ensure the transformation is intentional, not varied based on personal characteristics or contexts, or accidental. Each birth is unique and complete. We have one opportunity to get it right, every day and every time, and you hold the keys to make these possible for all women and families. Three things I'm asking of you today: awaken your childlike / professional curiosity to see what you can learn and consider changes you might have to make when discussions get uncomfortable and even challenging. Do not be satisfied with easy answers. Enable the full funding of MVPs and the National Maternity Voices that represents, supports, and networks their chairs… Resourcing the National Maternity Voices and MVPs may be the most cost-effective thing the NHS can do to improve maternity care because it enables meaningful co-production with service users (…)*.

Delivered by Mo, an MVP Chair, at the NHS Maternity Summit on 6th May 2021.

The extract presented above, from the opening remarks of the NHS National Maternity Summit in May 2021, delivered by the Patient Leader and MVP Chair, Mo (also co-author of this study), a mother who lives in South East England, demonstrates the foundational elements of the Public Narrative framework. First, Mo grounds her remarks in the specifics of her own lived experience as a woman, an expectant mother and a service user (Mo's **“Story of Self”**). Second, Mo creates moments that resonate with other women and families, while also speaking to a more expansive community (clinicians; managers; NHS regional and national leaders) who share the responsibility to “get it right” for women and babies (Mo's **“Story of Us”**). Third, Mo makes specific “asks” of that community (i.e., to fully resource the National Maternity Voices and the Maternity Voices Partnerships (MVPs) in a compelling, credible way by calling on that community to recognize this challenge as both important and urgent (Mo's **“Story of Now”**).

Public Narrative is a framework that links these three elements - Self, Us, Now - into a single practice for articulating stories of change (1). As one Patient Leader from within a cohort based in South East England, Mo spent several months exploring and practicing Public Narrative approaches within her work as the Chair of her local MVP. While research has been undertaken on the use of Public Narrative within community organizing contexts (2), more can be learned about how this framework for public leadership can be adapted effectively in the context of public service more broadly and healthcare and maternity services in particular.

Leadership is ultimately a process of constructing and coordinating a shared and dynamic sense of “us,” negotiated inter-personally and subject to change (3). Leaders work to forge a stronger sense of commonality, often using shared experiences as a basis to develop narratives of shared identity with others. Yet while this practice of leadership is widely acknowledged (4), relatively little attention is given to describing the methods and approaches which build the confidence, skills and capacity of leaders to develop it. Rather, the technical skills of efficient management (using cognitive skills, the “head”) are given preference over the intentional cultivation of the adaptive skills that enable leaders to develop empathic connections (the “heart”) and more purposeful and strategic collective action (the “hands”) (1).

A turn should be made to capitalize on the leadership and the opportunities offered day after day by the “lived stories” that all leaders have which are constantly in flux, being reshaped, told and re-told with new episodes and new meanings (5). Stories have a great potential to extract knowledge for the practice of leadership if cultivated, allowing leaders to learn with intentionality and purpose, and being sources of meaning, thus better preparing them to inform future horizons, and face the “unknowns” that can emerge (6). This way, in this study we analyse a pilot initiative conducted with one cohort of Patient Leaders, the Chairs of local MVPs in South East of England, and how they have used the Public Narrative framework to enhance their effectiveness in leading transformation in maternity services as part of the NHS Maternity Transformation Programme. Grounded on existing research about the key role of storytelling for leadership practice, and more specifically, on the potential of using it in the field of maternity services, the study of this pilot initiative conducted in England indicates that the Public Narrative framework provides a key method to support public service leaders to develop the skills, confidence and capacity to lead transformation more effectively.

### The maternity voices partnership, leadership, and the use of Public Narrative

#### The context of maternity voices partnerships and maternity transformation

The MVPs are an independent NHS working group established to champion the voices of women and their families in the development of maternity services in England. The MVPs aim to improve people's experiences of maternity services through multi-disciplinary collaboration and co-production, bringing service user voices to the center of planning and strategy. Within this mission, the elected and recruited Chairs of the MVPs play a crucial leadership role, both as Patient Leaders in their own right and as lead representatives within their localities, “bridging the gap” between service-users and healthcare professionals.

The context of the MVP Chairs' work is challenging. In December 2020 the Ockenden Review, an independent review of maternity services at Shrewsbury and Telford Hospital NHS Trust (7), published its interim findings. This built on earlier reports that identified significant needs for improvement in maternity services within the NHS in England (8, 9). The Ockenden Review highlights ongoing challenges to ensure that NHS maternity services are delivered in a consistently safe and personalized way for all women and babies. The “immediate and essential actions” outlined in the interim findings emphasize the need for all those responsible for supporting maternity services in England to attend to the culture and leadership elements of service improvement. This emphasis recognizes the interlocking set of roles, processes, values, beliefs, attitudes and assumptions that enable (or inhibit) safe and personalized care, including the critical contribution of Patient Leaders.

Beyond challenges specific to maternity services, the national leadership of the NHS in England has set ambitious aims for developing a more inclusive and responsive overall healthcare system (10, 11). These aims recognize that persistent, structural inequalities in service design and delivery remain. They make reference to the negative impact of persistent inequalities on marginalized and vulnerable people (12–14). Meanwhile, the ongoing impact of the COVID-19 pandemic continues to expose the variable impact of health inequalities and the urgent need to further develop healthcare leadership in the NHS (15, 16).

As NHS leaders seek to address these challenges, renewed emphasis is being placed on integrating the perspectives of Patient Leaders into service design and delivery. A range of national arms-length and professional bodies have taken steps to implement the Ockenden Review's interim findings, which explicitly recommend listening to women and families. The NHS Operational Planning Guidance cites a multi-million-pound boost to support collaborative delivery of these improvements in maternity care (10). Together, these steps represent a significant shift away from preferencing action “above the waterline” – incentives, structures, policies, goals, regulations and so on – toward an emphasis on the cultural elements “below the waterline” – narratives, beliefs, understandings, shared assumptions, judgements, values, unwritten rules, myths, and how things *really* get done (17, 18).

#### Integrating the voices of service-users into maternity service transformation

Abundant literature explores the role of patient participation as a key component in the (re)design of health care processes (19) and the positive impact of patient participation on patient experience and outcomes (20–22).

As it relates to maternity services, most research focuses on patient engagement and shared decision-making during maternity care (23–25), as well as the benefit of prioritizing patient voice and patient experience (26, 27). Studies demonstrate the value that women place on good communication, high-quality information and being supported to have a sense of control (28). Existing research further shows the reciprocal value that clinicians place on acknowledging patients' experiences and actively considering patient wishes in their work (29).

Good quality communication, complemented by the active involvement of service-users, are consistent features of those hospitals which deliver safe and personalized care during childbirth. In a qualitative study conducted by Liberati and colleagues (18), the authors identified seven features of safety in maternity units, with an emphasis on collegiate behaviors that value expertise rather than hierarchy and the active inclusion of patient perspectives. Further, major policy directives within the NHS in England set out a clear commitment to actively incorporate the perspectives of service-users (10, 11, 30).

Yet while all seven features of safety identified by Liberati and colleagues require collaborative and inclusive practices to be fully realized, extensive evidence has documented how hierarchical relations within the healthcare sector, and in maternity services specifically, continue to impact negatively on both patient experience and outcomes (31–36). Practices which permit and/or reinforce these hierarchical relations are known to support implicit bias and interpersonal racism and sexism within maternity services, with serious consequences for the mothers and babies affected (37–40).

Within this context, we explore how the Public Narrative framework may be used to enhance the effectiveness of Patient Leaders in maternity services to influence positively for change. We do this by conducting a case study which examines how the Public Narrative framework has been used with and by a cohort of Chairs of MVPs in the South East England, by means of a pilot training and coaching initiative commissioned by NHS England and NHS Improvement – South East.

#### The Public Narrative framework as a model for leadership practice

The Public Narrative framework is a model for leadership practice. It describes an approach to developing leadership skills, predicated on the basis that “leadership” is the action of accepting responsibility for enabling others to achieve shared purpose under conditions of uncertainty (1).

The Public Narrative framework supports the ability of participants to uncover, articulate and then iterate narratives purposefully and self-reflexively, drawing on the resources of their own lived experiences (and those of others) to create possibilities for collective action. Public Narrative is grounded in the narrative capacity of human beings to identify, interpret and then construct our own social reality in negotiation with others (41). As Ganz explains, crafting a Public Narrative involves unearthing lived experience and articulating it using three elements: a “story of self,” a “story of us” and a “story of now.”

A **story of self** is a narrative account of the basis on which I, as an individual, have been called to lead. Stories of self-articulate specific moments, choices and challenges within a person's lived experience that reveal something of the person's underlying values.

A **story of us** expands a person's narrative account to encompass a wider constituency of people with common experiences, concerns and/or underlying values. Stories of us aim to engage the listener and activate a shared sense of purpose within the overall narrative (42).

**Figure 1:**
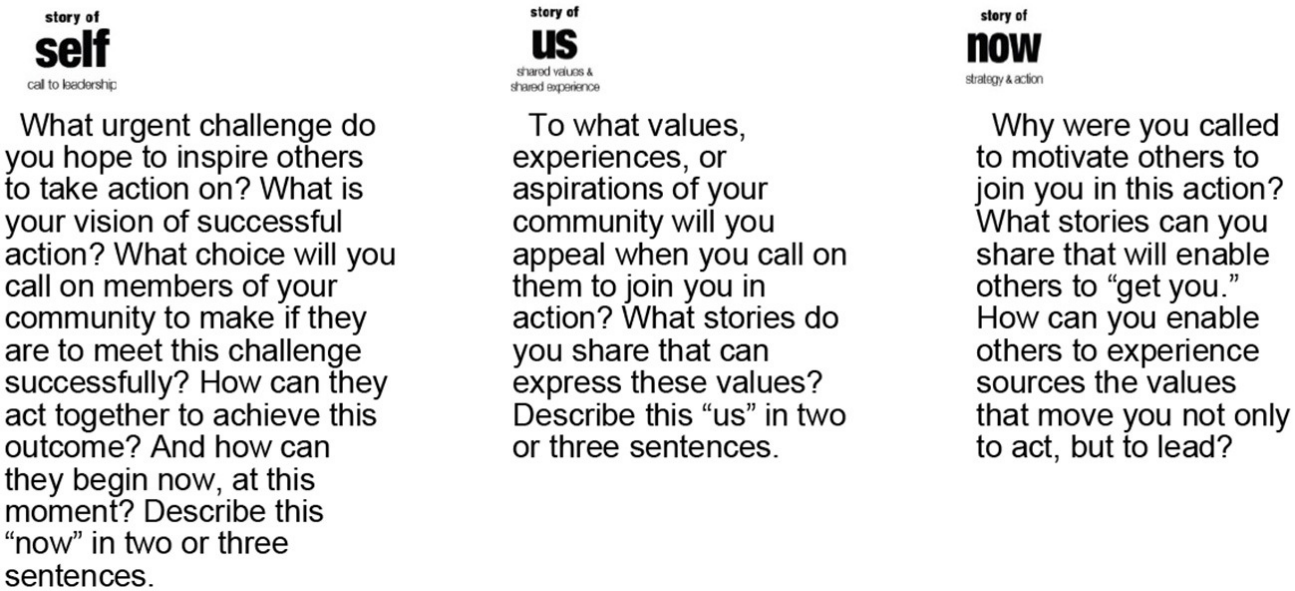
What is Public Narrative: Self, us & now.

**Figure 2:**
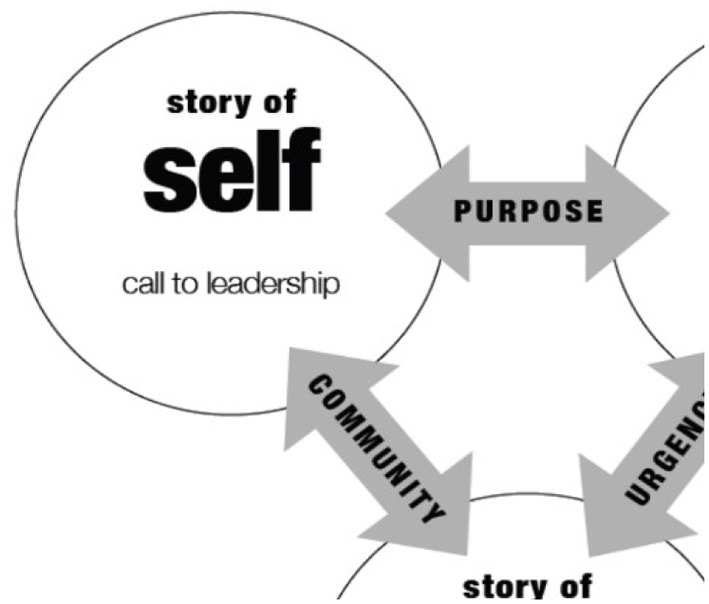
The three elements of Public Narrative. Source: Marshall Ganz. 2009. What Is Public Narrative: Self, us & now. (Public Narrative Worksheet). Working Paper.

A **story of now** links the narrative account to a real and urgent concern or opportunity, which the constituents could influence by acting together. Stories of now articulate specific requests of the constituents, and/or they unite them around specific requests that they must make of others. Stories of now aim to activate the listener's sense of agency, describing a hopeful vision of what intentional, collective action can achieve. Figures 1, 2 below showcase the three elements of Public Narrative: self, us, and now.

The dialogic-centered pedagogy (43, 44) for the learning and crafting of Public Narrative makes this process inherently relational, experiential and reflexive (4).

First, Public Narrative is embedded in a relational mode of leadership action (45). The framework is learned, modeled and practiced inter-relationally with others. Second, as Ganz (1) summarizes, the experiential basis of the pedagogy of Public Narrative situates it is a means of learning how to lead, not simply a framework for describing leadership. Learning occurs both through the practice of Public Narrative and through reflection on that practice. The component elements of *story of self, story of us, and story of now* are used in real-time situations to develop leadership capacity. Third, the learning process of Public Narrative is reflexive as well as relational and experiential. It involves an ongoing process of the individual reflecting upon their/her/his lived experience (story of self), and then seeking to understand it in relation to the experience of others (story of us and story of now). In this way, a person's capacity to be both an individual and part of a group, using both states of being to lead more effectively, becomes possible. Thus, Public Narrative is not a formula for how to lead but rather a framework that guides the individual and the group to ask questions which resonate both individually and collectively, with the potential to activate their sense of shared purpose. The construction of different narrative moments (46) about the self, us and now enables participants to assemble coherent and compelling stories for change.

Marshall Ganz and his collaborators began to develop a pedagogy of this practice in 2006 and have adapted it since in online and offline courses, workshops and projects based at Harvard University's Kennedy School of Government. Between 2006 to 2016, more than 32,000 people participated in 448 workshops in some 25 countries including Denmark, Serbia, Jordan, India, Vietnam, China, Japan, Australia, and Mexico, and in domains as distinct as healthcare, education, politics, religion and civic advocacy (47).

In this study, we explore the use of the Public Narrative framework as a model for leadership practice in the context of the Maternity Voices Partnerships in South East England.

## Methods

This case study was conducted within the broader Narratives4Change research project (2019-2022) funded by the European Union's Horizon 2020 program (grant agreement No 841355) and led at Harvard Kennedy School and at the Department of Sociology of the Autonomous University of Barcelona (UAB). The case study was developed in two phases, Phase 1 consisted in the implementation of the pilot intervention on Public Narrative learning sessions and further coaching support to enable real-world delivery of the developed skills. The approach in Phase 1 was co-designed, modeled and delivered with Patient Leaders throughout the whole process of the pilot initiative. Phase 2 consisted of qualitative data collection conducted between January and April 2021, undertaken during and after the learning sessions and further coaching, as well as the subsequent process of data analysis. Besides, an additional and complementary round of qualitative data collection was conducted between July and August 2022 aimed at contrasting initial data and exploring the sustainability of the impact achieved by the intervention. Below both phases are briefly described.

### Phase 1. Public narrative training sessions

In July 2019, NHS Horizons, a small national team within the NHS in England with expertise in large-scale change methods and practices, was commissioned by the Maternity Transformation Programme in South East England to lead two full-day workshops using the Public Narrative framework. The first workshop, co-designed with a Regional Chief Midwife, was delivered in September 2019 to a cohort of some 30 senior practicing midwives. The second workshop, co-designed with a senior practicing midwife trained in the Public Narrative framework, was delivered in March 2020 to a cohort of some 50 practicing midwives.

As a result of these workshops, in June 2020 NHS Horizons was commissioned to co-design and lead a pilot initiative that included a more involved series of training and coaching with MVP Chairs across South East England. Table 1 below summarizes the staging of the co-design and implementation:

**Table 1:** Phase 1: Co-design, co-facilitation, and delivery of Public Narrative.

Step 1. Pilot initiative launch: co-design, group constitution and introduction of Public Narrative to the MVP co-design cohort	Formed a co-design group and held three x 90 min/2-h sessions in which the Lead Coach taught using the Public Narrative framework. The co-design group comprised 4 MVP chairs (former and current) and 1 stakeholder of the Maternity Transformation Programme.
Step 2. Coaching and follow-up with the pilot co-design group	Once the co-design group was constituted and had been introduced to the Public Narrative framework, a training session design was drafted and then adapted in co-production with the group to meet the needs and context of MVP Chairs.
	Meanwhile, the Lead Coach conducted individual co-design and coaching sessions with two participants in advance of the two x 2-h Public Narrative sessions with MVP Chairs. The two participants continued to practice and refine their Public Narratives and developed the relationships and understanding to co-facilitate the subsequent two x 2-h Public Narrative sessions.
Step 3. Delivery of two Public Narrative sessions to the larger group of MVP Chairs	In the two x 2-h Public Narrative sessions with MVP Chairs, approximately half the participants had received prior coaching in Public Narrative (steps 1 and 2) and half were participating for the first time. The sessions aimed to create a sense of team (“us”) between both types of participants. During the sessions, some participants described how the sessions were the first occasion on which they had, as a cohort, worked intentionally together on their relationships, to better understand their shared aims and goals (purpose) and their potential to influence collectively as MVP Chairs. 9 participants joined the first session; and 12 joined the second one.
Step 4. Debriefing	After the two x 2-h Public Narrative sessions, a separate debrief session was convened with the co-design group.
Step 5. Ongoing support	The Lead Coach provided ongoing support to members of the co-design group. One participant recorded her Public Narrative for the (virtual) School for Change Agents 2021, launched in May 2021 with 4,500 participants (available online) (48). As part of the School for Change Agents, that participant also recorded a video to describe her past experiences of trying to make change happen and how Public Narrative training and coaching had enhanced her effectiveness as a MVP Chair.
Step 6. Deployment of Public Narrative work public, decision-making spaces.	An MVP Chair who took part in the pilot initiative presented her Public Narrative at the outset of the NHS National Maternity Summit, which brought together the most senior leaders of national bodies whose work impacts on maternity services under the leadership of Ruth May, Chief Nursing Officer, and Jacqueline Dunkley-Bent, Chief Midwifery Officer, in May 2021.
	The Summit represented a milestone opportunity to catalyze a “significant, stepped improvement” (Ruth May, CNO) in how NHS leaders better align their current and future work at a national level to support maternity services in a highly joined-up and effective way.

**Figure 3:**
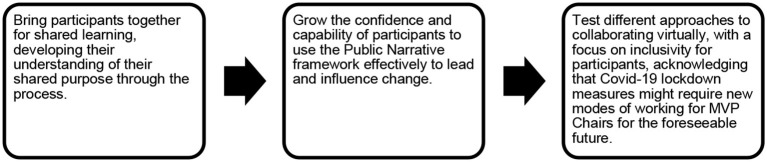
Public Narrative pilot training aims.

The MVP Chairs participating in the initiative varied widely in terms of their length of experience in-role, as well as their underpinning experience and confidence to lead and influence change. Figure 3 below shows the aims agreed for the pilot initiative.

By way of follow-up, individual MVP Chairs were then coached and supported to use the Public Narrative framework to tailor their contributions to public-facing work, culminating in the delivery of one participant's Public Narrative as a whole-room contribution to inaugurate the national NHS Maternity Summit in May 2021.

### Phase 2. Qualitative data collection and data analysis

Qualitative online fieldwork was conducted between January and April 2021. Three data collection techniques were used:

1) Participant observations of the two learning sessions of the intervention (described above), which were agreed not to be transcribed. Instead, notes of the dialogue, the interactions, reactions, questions and challenges posed were taken.
2) Focus groups. All MVP Chairs who had participated in the two training sessions were asked to voluntarily participate in two focus groups aimed at exploring their views on the Public Narrative training; if (and, if so, how) they have used it since; and their perception as to its relevance (or not) to their work as MVP Chairs.
3) Semi-structured interviews with key stakeholders and members of the co-design group. Semi-structured interviews were conducted with 3 key stakeholders: 2 senior midwives with management roles within maternity services and 1 stakeholder with a leading role in the national Maternity Transformation Programme. Besides, 4 semi-structured interviews were conducted with MVP Chairs who had joined the intervention.

Between July and August 2022 an additional round of qualitative fieldwork was conducted with the intention to contrast and revisit initial data gathered, and also to explore the impact of the intervention in the subsequent 15 months since the initial training. During this period and because of the initial training and coaching initiative, at the beginning of 2022 a second initiative emerged, the “Stories for Change.” This was commissioned by the same funder and put in place as a collaboration between NHS Horizon and the MVP Chair (involved in the previous training), regional service user representative for Public Narrative. The service user role was created by NHS England South East to support a service user centered embedding of Public Narrative as part of their maternity transformation programme's perinatal equity work. The details of the “Stories for Change” project was co-designed with maternity service users who initially responded to adverts that were disseminated through the MVPs in the South East. In it, the Public Narrative framework was used as a way to empower women to use their maternity experience as a catalyst for positive changes in their local maternity service., They were supported to craft their stories for change in a way that challenged health care professionals to consider more effective wayss to work together to make maternity care more equitable for mothers and babies from diverse backgrounds, particularly those from ethnic minority backgrounds and those living in deprived areas.

Interviews were conducted with 4 women who are either part of the previous Public Narrative offer to MVP Chairs or members of the “Stories for Change” co-design group: 1 MVP Chair from the MVP Chair training cohort and 3 service users from the “Stories for Change” project, one of whom is also a Vice Chair of an MVP. Table 2 below summarizes qualitative data gathered conducted for this case study.

**Table 2:** Summary of qualitative data collected.

**Technique**	**Number of participants**	**When**
2 Participant observations of the two learning sessions of the intervention	1 x 9 participants	November 2020
	1 x 12 participants	
2 Focus groups	1 x 4 participants	January/April 2021
	1 x 4 participants	
11 Semi-structured interviews	3 stakeholders: 2 senior midwives with management roles within maternity services and 1 stakeholder with a leading role in the national Maternity Transformation Programme	January/April 2021
	4 x MVP Chairs who had joined the intervention	
	1 x MVP Chair member from the previous intervention group	July and August 2022
	1 x MVP Vice Chairs member of the co-design group of the with an active role in the “Stories for Change” initiative	
	2 x Service user and member of the co-design group of the Stories for Change initiative	

In all, the total number of participants in this case study is 28 women, excluding replication of the ones interviewed who had also been in the two learning sessions and the focus groups. This includes users of maternity services, MVP chairs, NHS stakeholders, and midwives.

### Data analysis

Solely the two focus groups and the semi-structured interviews were recorded and transcribed. Notes were taken about the insights of the session and the debriefing with the participants. Notes were also taken by co-author 1 from her observation of the two training sessions. All qualitative data gathered from Phase 1 and Phase 2 was coded and analyzed using NVivo software (Version 12). An initial coding scheme was created drawing on the Narratives4Change project general research questions, and the specific objective defined for this study: *in which ways can Public Narrative be used by MVP chairs, and advance their leadership within maternity services?* In turn, the coding process followed comparative analysis guidelines used in grounded theory.

Cross-cutting issues explored and looked were the impact of Public Narrative on the following aspects: communicate why I have been called to leadership; understand values shared by others; encourage individuals to feel confident about expressing their vulnerabilities; build mutual understanding; build trust within a group; facilitate agreement on a shared purpose; facilitate a sense of cohesion within the group; define what the “ask” is; communicate urgent needs and opportunities to others; inspire hope that the action would make a meaningful difference. Co-authors engaged in a dialogic process at the time of analyzing and interpreting the data, triangulating interpretation of results with fieldwork participants.

## Results

In this section we present the findings of our study, which are structured in three main sub-sections. First, we explain how the idea emerged to use the Public Narrative framework with MVP Chairs, such that the motivation and rationale for the intervention are established. The second and third sub-sections set out the core findings, framed using the MVP Chairs' own voices as captured through qualitative fieldwork done in 2021 and in 2022.

***“We've the structure, but we need the values:” the motivations and rationale for using the Public Narrative framework with MVP Chairs***

In late 2019, the South East Regional Team of the national NHS Maternity Transformation Programme commissioned NHS Horizons to co-design and lead the delivery of a leadership development offer for MVP Chairs in their region. This commission followed the successful co-design and delivery of similar offers with cohorts of senior maternity professionals and practicing midwives, which had focused on creating shared purpose within the cohorts and growing the individual participants' confidence and skills to use narrative-based approaches to change.

Midwives interviewed for this study, who participated in the earlier co-design and delivery offers, identified two opportunities to expand that work into an offer for MVP Chairs. First, they identified the potential for Public Narrative training to create more of a sense of “us” between the MVP Chairs, deepening their relationships to enable more effective collective action in future. Second, they identified the Public Narrative framework's potential to provide a structure for MVP Chairs to develop their skills as advocates whose role is to promote patient involvement in and coproduction of maternity services.

“The [Maternity Transformation Programme] it's about safety, it's about being more personalized and being kinder, and providing choice for women, and starting to get on a level playing field with the women that use our services so that their voices are equal (…). And we've got the structure, but what we need is actually those values to live.” (Senior midwife, Stakeholder 1).

Professionals interviewed explained that fostering a more inclusive culture in maternity services requires reciprocal communication between different healthcare professionals, as well as with patients, to establish and grow trust. They described the importance of developing MVP Chairs' skills and confidence to use narrative approaches to influence others, especially clinicians:

“So the difficult part is to move on professionals from hearing women's feedback and their stories to absolutely embracing women as equal partners in the design of maternity services and we're still on a journey there (…)” (Senior midwife, Stakeholder 1).

A common theme in the interviews was the need to “bring the heart” into how MVP Chairs think about their roles, as a complement to the structural and governance aspects of changes which may be viewed as predominantly based on “head” (strategy) and “hands” (action). Midwife 2, reflecting on her own experience as a participant in the earlier co-design and delivery offers, stated the need “to transform maternity services from within:”

(…) we know we need to transform maternity services. But people haven't felt involved in the process … And this type of collaboration [i.e., the Public Narrative offer] makes you feel like you are all in this together (…) I've had loads of conversations with people, other midwives, emails, messages… saying: “you know, I was really thinking about what you were saying”… and that sort of thing you don't necessarily get if you are going in telling people what they need to do (…) (Senior midwife, Stakeholder 2).

As stated by the NHS People Plan 2020/2021 (11) and emphasized by all interviewees, one of the biggest challenges in England in maternity services is to ensure equity for mothers and babies from Black, Asian and Mixed ethnic groups and those living in the most deprived areas. As such, the Public Narrative offer with the MVP Chairs aimed to complement this on-going process of structural change taking place in maternity services, by focusing on the MVP Chairs' shared purpose and their skills for influencing change. This way, the use of Public Narrative in maternity services with both health professionals and service-users shows potential to close the gap between the two ends, professionals and service users. This was well-elaborated and explained by one study participant, a member of the co-design group of the Stories for Change intervention, from an ethnic minority background, who is serving now as MVP. She emphasized the potential of using Public Narrative as a way to get the stories of those most-hard-to reach mothers, due to their ethnic and socio-economic conditions, to be heard:

“And I think that the reason why the training of Public Narrative that we went through really helped was because I had been part of the co-design group and had listened to other stories and felt the power of stories myself, ending in tears many times… Because as a mother yourself you feel it, you feel for the other women, especially if you have been through something similar (…). So we have to learn from these stories, we have to do something about them, and we have to action (…). And I think that those are crucial to improve things in the Maternity Services. Obviously, the current narrative within maternity is that ethnic minority groups are not treated the same as their white counterparts and that is where a lot of the work that I have been doing comes in… However, is much bigger than that, it's just about every single woman being able to have the opportunity to have a birthing experience that just is not solely trauma (…)” (SFC 1).

### Articulating “Our Story” in a way that forges shared purpose

Stakeholder 3 interviewed for this study has significant experience overseeing, planning and supporting the development of the Maternity Voices Partnerships in England. She emphasized the potential of the Public Narrative framework to deepen relationships between MVP Chairs, as a counterweight to their acquisition of the technical competencies required to be effective Chairs:

“People can relate to stories (…). Everybody can be a good narrator, but we need them to provide a framework. I pretty much appreciated the idea that the training was co-produced and co-designed with MVP Chairs, was tailored with them. So MVP Chairs were really inspired. (…). I think that Public Narrative is a way to facilitate this change but not in a technical way… no no, it's not about that but about our shared mission” (Stakeholder 3, Maternity Transformation Programme).

During interviews, several MVP Chairs and stakeholders described how the Public Narrative framework has enabled them to reflect more deeply on their own values, including their individual motivations for committing time and energy to the development of Maternity Voices Partnerships. One MVP Chair linked this to the wider need to establish connecting ties, or an “empathetic bridge,” between MVP Chairs:

“[E]ven in our differences within the groups, we must not forget that our common humanity as a common ground, you know, … This idea that because somebody has a totally different experience than yours so therefore they cannot connect with ours. Yes they can. I think we must challenge it (…). We mustn't forget that it's that common ground that galvanizes us, because we need each other (…) (MVP D).

Several interviewees described how the connection through values that the Public Narrative offer enabled supported better communication between MVP Chairs both during and after the training and coaching sessions:

“The whole purpose of this is to pull people back to their core values, to really remember why we're here. And I think if people can tune into that, to me it's not always about what they're saying out loud, it's the preparatory work that they've done to tune into their core values that then will empower them, and their Public Narrative still might not be fantastic… they still might not feel compelling influencing the director of finance that you need more money, but actually they tuned into their core values and then that will realign their service” (Senior midwife, Stakeholder 1).

The importance and need of improving communication between service users and maternity care medical staff was also highlighted by the NHS staff that were interviewed for this study. They highlighted how Public Narrative helps to improve communication between healthcare providers and service users. A service user participant in the Stories for Change initiative shared an example that illustrates this:

“I shared parts of my story using the Public Narrative structure during the break out rooms of the Stories4Change Event, I was surprised at how this led to each and every other member of that room sharing parts of their own stories. In the past when I've shared my story (prior to using PN) I've often been met with either silence or comments that felt quite dismissive such as making excuses for services, telling me it could have been a lot worse etc. This time round, even though the participants were mainly midwives they did not respond with defensiveness or excuses but opened their hearts to share experiences that they too had felt hurt by that were very similar to mine” (SFC 2).

Midwife 2 further highlighted the prevalence of informal storytelling within maternity services but noted that it is neither recognized nor explicitly valued as a leadership practice. She described the value of Public Narrative as bringing structure and intentionality to storytelling, such that it becomes a skill that leaders can hone with others:

“Telling stories is something that we do very well as health professionals and we have a lot of stories in our bank because we meet so many people (…). And I always thought I could just chat and it was good to be able to talk to people. But no, actually… to be able to use those stories to actually bring people with you is what… There has to be an additional component, that's what I have taken out from the Public Narrative framework.” (Senior Midwife, Stakeholder 2).

These observations indicate that the Public Narrative framework offers a scaffolding for participants to structure their narratives in ways which both enable deeper personal reflection and provide the basis for them to develop meaningful relationships as peers. This was shared by both the MVP Chairs that were interviewed as well as by members of the Stories for Change initiative:

“I think that day to day I do stories like the Public Narrative message… I think that I'm more conscious of that now that I have done all the Public Narrative thing. But pride to that is the way I felt that I was bonding with the moms around me…. Talking to strangers… It makes you more competent when I am talking to people knowing that we all have a story that is worthy of being listened to” (SFC 2).

Several interviewees also identified a link between the specific need for MVP Chairs to develop their shared purpose and the broader need for more trusting relationships to be forged between patients and healthcare professionals:

“The sad reality when it comes to families is that it's always the same “I didn't feel listened to”… The parent does not want to say who it was… So this is why I got involved.” (MVP Chair C).

In this way, evidence gathered suggests that the Public Narrative framework may support relationships to develop *within* a cohort and may also prompt further thinking on the importance of developing relationships more widely. In particular, Public Narrative may offer one means by which service-users can develop the skills and confidence to support healthcare professionals to listen to them, building trusting relationships that encourage potential health problems to be shared and addressed before they progress in severity. For example, MVP Chair C shared how participating in the Public Narrative offer had prompted her to re-assess how she works with volunteer service-users whose role is to encourage healthcare professionals to listen to women and their families:

“All of us have a story to tell, and we want to share that. And I need to create the space for my volunteers to share that… Because If I do that, they will listen more carefully to parents that come to the service (…) If I'm interviewing someone I'll ask the person, what's your experience with maternity… some of them have had an awful experience… and we need to go over that in order to make sure that we are doing a good job” (…). And even more, if you are a parent it's very difficult to speak up, if you are going to have a baby you are afraid of midwifes… (MVP Chair C).

MVP Chair E articulated how working on a “Story of Us” led her to better understand the ways in which service-users and healthcare professionals are “one team.” This, in turn, has the potential to reinforce bonding ties that cross sectoral and role-based identities, creating a more expansive common purpose shared by those who both provide and use maternity services:

“I don't think that nobody goes into midwifery thinking “I'd really like to go in there dismissing the experiences of service users…” I mean, making service users feel rushed, making them feel just a number… Having that compassion for staff … is that other “Story of Us.” [It is] such a powerful thing to be able to do because then you really are bringing together that with you and that leads you into the Story of Now, about what we can do.” (MVP Chair E).

The potential of the Public Narrative framework to expand participants' sense of their options, in terms of their own field of influence as leaders, was alluded to by several MVP Chairs:

“I think that the Public Narrative framework is a very hopeful framework, one that is focused on progress (…). This is not just about your story, or even sometimes that self-indulgence triumph of your story, no. It's to kind of go out and see a bigger picture. It takes you out of you to see the bigger picture (…) (MVP D).

As such, evidence gathered suggests that the use of the Public Narrative may both deepen relationships *within* a cohort and prompt further thinking on the importance of developing relationships more widely. Central to this process is the negotiation between participants of what they hold in common (stories of us) and how those experiences and values relate to their work together – their shared purpose.

### A structured way to communicate shared values: Developing the skills, confidence, and capability of healthcare leaders

“I have found Public Narrative useful not just within MVP, but also personally. It gives you a structure about how to think. For someone like me… I've got loads of thoughts a lot of the time… My head is full of ideas with so much. I think Public Narrative helps you to calm down and think with others, with your team, with your colleagues: Okay, what story are you trying to explain? How does this connect with other people? How can you link this with others' people story?” (Focus group, MVP D).

“I've been given not only the time, but also the kind of parameters in which to really think about why I did this role, what we wanted to sort of do as an MVP [Chair].” (Focus group, MVP F).

Several interviewees described how participating in the Public Narrative offer had provide the time and space to think differently about their motivations as MVP Chairs and as maternity service users within a structure framework for learning. Our study suggests that such facilitated spaces enable Patient Leaders to prepare for their role as both decision-makers and influencers working alongside healthcare professionals who hold hierarchical positions of power within maternity services:

“That's where I felt that Public Narrative was really important, that the women would be able to articulate exactly what they wanted and to have a conversation at executive level. Because quite often they're dealing with a very hierarchical NHS. And of course, women can feel very small, and so it was to try and give them a tool to enable them to tell their story in a way that was positive and actually achieved what we wanted to achieve. In our initial trainings it was evident that there were blockers for the clinicians, for the midwives around influencing executive level, but also influencing down into their staff groups and talking about the change in an engaging and positive way.” (Midwife, stakeholder 1).

MVP Chairs who were interviewed echoed these observations. They described the NHS in terms of hierarchical authority, presenting significant challenges for Patient Leaders seeking to navigate the decision-making structures to influence change:

“This is a very emotional job… from where people come to me as a MVP Chair… so I would say that if I feel that my role is to support the service users, the families accessing the services are not necessarily to appease the NHS or the local trust (…) So it requires having that balance of being able to serve the service users, which is what I feel is my job… and navigate the NHS bureaucracy.” (Focus group, MVP A).

Within this context, several MVP Chairs including the 4 interviewees who were interviewed months after the pilot initiative (in July and August 2022) referred to the importance of the Public Narrative framework providing a structured approach to articulate and communicate their thoughts and ideas. Interviewees described the dynamic shaping of stories (blending the elements of self, us and now) within a structured framework that can be “taught” as crucial to creating authentic and compelling stories for change. An example illustrating this was explained by one of the interviewees who is part of the Stories for Change initiative; she explains how Public Narrative gave her a structure to order and express her thoughts, and how when she delivered it in a room full of medical professionals, there was a shift in the way the problem was being approached:

“Using PN has given me both a structure and focus that has naturally become part of how I now frame and present things, it has helped me be brave and it has helped me gain resilience (…). The first meeting I went to after the project was a meeting where I felt completely out of my depth. I was the only service user in attendance amongst senior medical staff. I was almost amazed at how when I got the courage to share (using PN) I felt an almost instant softening of the atmosphere in the room. There was an instant shift in the direction of the problem solving that had been occurring and the room realized that the problem we were trying to address actually needed to be addressed from a different direction” (SFC 1).

Besides, interviewees emphasized the difference, in this respect, between Public Narrative and public-speaking or presentational skills: the former builds skills and confidence to craft and then adapt authentic stories over time and in relationship with others. Our study suggests that the collective sense-making element of the Public Narrative offer was crucial, providing a structured way for the MVP Chairs to communicate their shared values:

“It's a way of putting it across … You can give your experience with everything else to help promote that change (…) You can gather the information and just explain it in a technical way, or you can make sense of it, and communicate that considering all your experience…: “Hey, this is what's happening, this is what people are telling us.” And Public Narrative can be intentionally used in that very aspect. So you're bringing it all together to say: “This is not just about me and my personal situation, but my personal situation is part of that…” (MVP A).

“Public Narrative has actually given me enough confidence to say “Yes. I'll be speaking in public.” But also it gives me a framework for how I might do that. Actually, it's kind of like “I get the point of what public speaking is, something on which I have neither thought nor felt before. It was just a horrible thing. I really struggle with it, “please don't ask me to do it,” you know, whereas this actually… It's there's a point to that. It's to inspire people. I never thought for a second I could inspire anyone to do anything.” (Focus group, MVP E).

Also, evidence collected suggests that using a structured approach, with a facilitative mode of delivery, enabled the Public Narrative offer to develop the skills, confidence and capability of MVP Chairs and service users to communicate their shared motivations and values, and to do so with more courage before medical staff. This was pointed out by most of the MVP Chairs interviewed, as well as by two members of the more recent Stories for Change initiative:

“[I]t gave me a validation to be able to get my story across in a way that did connect with other people because I'm aware that the MVP is not about our personal stories, or about the one issue or two issues.” (Focus group, MVP F).

“It's made me more self aware of how I am feeling in any given situation, especially when I am collecting feedback from people. It's meant I'm much more likely to share something about myself, my values and feelings to identify with the person I am talking to whilst being careful to keep the focus on them and not making it all about me.” (MVP & SFC4).

The Public Narrative offer further supported the MVP Chairs to cultivate compelling narratives based in their lived experience, which they could then use as “resources” in their real-world work. Our research indicates that the use of Public Narrative may serve to validate the implicit knowledge and expertise of Patient Leaders by re-connecting them with the underlying value of the experiences that initially prompted them to take on their public roles.

## Discussion and conclusion

While evidence points toward a shift in good leadership practice away from “command and control” styles toward an approach rooted in influence, motivation and shared purpose (49–51), there is limited research into the most effective frameworks, methods and practice that enables this shift to happen.

Both the legislative and policy agendas of healthcare in the UK place emphasis on this shift, including the need for more collaborative, person-centered approaches to the design and delivery of services (10, 11, 52). Yet we currently lack a coherent understanding of the process to implement the changes this requires.

Extensive research shows that good communication is central to the design and delivery of safe, personalized care in maternity services (18, 23, 28). Yet relatively little support is offered currently by the NHS to enable Patient Leaders (and service-users in general) to develop the confidence, capability and skills to participate fully and equally in that co-design and delivery. Numerous NHS service reviews highlight the lack of effective inclusion based on the perspectives of women and their families as a critical risk in maternity services that must be addressed (7). These same reviews highlight the ongoing challenges that Patient Leaders (such as MVP Chairs) and service-users face in exerting individual and collective influence within the complex and hierarchical structures of the healthcare system.

In this case study we have presented and discussed the results of a pilot initiative that used the Public Narrative framework to develop the confidence, capability and skills of MVP Chairs in South East England. Our qualitative data reveals two main ways in which this framework for public leadership can enhance effective leadership within maternity services. First, we showed the ways in which the Public Narrative framework may support Patient Leaders to articulate “stories of us” such as to forge shared purpose within a cohort. Within this, we described the potential of the Public Narrative framework to deepen relationships between MVP Chairs, as a counterweight to their acquisition of the technical competencies required to be effective Chairs. Second, we provided evidence on the potential of the Public Narrative framework to offer a structure for Patient Leaders to craft dynamic and compelling stories which both communicate shared values and influence for change. Thus, Public Narrative offers a guided framework to use stories as key sources for organizational knowing, giving leaders those skills needed to be better equipped to adapt more rapidly to those changes that might occur in a dynamic future (5).

As such, we offer this case study as an example of the type of structured approach, co-designed and delivered facilitatively, which can make a significant contribution to realizing the policy aims of collaborative and inclusive service design and delivery set out above. Emerging research which identifies the behaviors and practices of safe care in hospital-based maternity units (“For Us” – For Units Safety) demonstrates that commitment to safety and improvement at all levels (with everyone involved), teamwork, cooperation, and positive working relationships are pivotal to reducing harm in maternity care and ensuring patient safety (18). Our study reveals one way in which these aims can be advanced more effectively with Patient Leaders.

The shift from more managerial ways of understanding leadership to those also focused on “accepting responsibility for enabling others to achieve shared purpose under conditions of uncertainty,” as understood in the Public Narrative pedagogy. It requires the commitment of those in managerial positions to take steps to involve end-users and constituents in the implementation of leadership changes. This study sheds light on how this can be implemented within large bureaucratic organizations like the NHS, in areas such as the maternity services, where existing literature shows the effectiveness of patient involvement in service provision. Advancement toward equity and equality in healthcare systems, particularly in maternity services, remains challenging, not solely in England but also across Europe. The use of storytelling for leadership, and in particular, of the Public Narrative pedagogy, provides an structured and guided framework to embrace leadership challenges and thus take action that engage with those cultural elements operating “below the waterline” in organizations, such as narratives, beliefs or shared assumptions (17). In all, the Public Narrative pedagogy shows potential to enable the accomplishment of the shift from transactional to transformational ways of understanding service users' relationships. It opes an avenue to make healthcare services, especially maternity services, more inclusive of the communities that are served.

Further investment in narrative approaches to change, and specifically in the co-design and facilitated delivery of learning initiatives using the Public Narrative framework, has the potential to foster a culture of inclusive, continuous improvement within maternity services and healthcare more widely. Further developing the confidence, capability and skills of MVP Chairs both to use Public Narrative and to facilitate its use by others would constitute a significant investment toward enhancing their effectiveness to lead positive transformation in NHS maternity services.

## Data availability statement

The datasets used and/or analyzed during the current study are available from the corresponding author on reasonable request.

## Ethics statement

The studies involving human participants were reviewed and approved by the Institutional Review Board (IRB) of the Harvard University-Area approved this study, IRB Registration Nr: IRB00000109. In addition, all information gathered for the Narratives4Change project complies with the Ethics Appraisal Procedure required by the Horizon 2020 research program, funded by the European Commission. Accordingly, Narratives4Change project follows the Regulation (EU) 2016/679, the EU new General Data Protection Regulation (GDPR). The patients/participants provided their written informed consent to participate in this study.

## Author contributions

EA and KP were involved in the conception of the study, designed, and contributed to the data interpretation. MA contributed to this analytical process ensuring that the views of patient leaders who participated in the study have been correctly understood and discussed. TS-M supervised and ensured the methodological quality and implementation of the whole Narratives4Change project in which this study is framed. All authors approve the final version of this manuscript, and all authors agree to be accountable for all aspects of the work.

## Funding

This study has been conducted under the framework of the Narratives4change research project, which has received funding from the European Union's Horizon 2020 research and innovation programme under the Marie Sklodowska-Curie Grant Agreement No. 841355.

## Conflict of interest

The authors declare that the research was conducted in the absence of any commercial or financial relationships that could be construed as a potential conflict of interest.

## Publisher's note

All claims expressed in this article are solely those of the authors and do not necessarily represent those of their affiliated organizations, or those of the publisher, the editors and the reviewers. Any product that may be evaluated in this article, or claim that may be made by its manufacturer, is not guaranteed or endorsed by the publisher.
